# Common variability in oestrogen-related genes and pancreatic ductal adenocarcinoma risk in women

**DOI:** 10.1038/s41598-022-22973-9

**Published:** 2022-10-27

**Authors:** Giulia Peduzzi, Livia Archibugi, Verena Katzke, Manuel Gentiluomo, Gabriele Capurso, Anna Caterina Milanetto, Maria Gazouli, Mara Goetz, Hermann Brenner, Roel C. H. Vermeulen, Renata Talar-Wojnarowska, Giuseppe Vanella, Francesca Tavano, Maurizio Lucchesi, Beatrice Mohelnikova-Duchonova, Xuechen Chen, Vytautas Kiudelis, Péter Hegyi, Martin Oliverius, Hannah Stocker, Caterina Stornello, Ludmila Vodickova, Pavel Souček, John P. Neoptolemos, Sabrina Gloria Giulia Testoni, Luca Morelli, Rita T. Lawlor, Daniela Basso, Jakob R. Izbicki, Stefano Ermini, Juozas Kupcinskas, Raffaele Pezzilli, Ugo Boggi, Hanneke W. M. van Laarhoven, Andrea Szentesi, Bálint Erőss, Giovanni Capretti, Ben Schöttker, Jurgita Skieceviciene, Mateus Nóbrega Aoki, Casper H. J. van Eijck, Giulia Martina Cavestro, Federico Canzian, Daniele Campa

**Affiliations:** 1 Department of Biology, University of Pisa, 56126 Pisa, Italy; 2 Digestive and Liver Disease Unit, Sant’Andrea Hospital, Rome, Italy; 3 Pancreato-Biliary Endoscopy and Endoscopic Ultrasound, Pancreas Translational and Clinical Research Center, San Raffaele Scientific Institute IRCCS, Milan, Italy; 4 Division of Cancer Epidemiology, German Cancer Research Center (DKFZ), Heidelberg, Germany; 5 Department DISCOG, University of Padova, Padua, Italy; 6 Laboratory of Biology, Medical School, National and Kapodistrian University of Athens, Athens, Greece; 7 Department of General, Visceral and Thoracic Surgery, University Medical Center Hamburg Eppendorf, Hamburg, Germany; 8 Division of Clinical Epidemiology and Aging Research, German Cancer Research Center (DKFZ), Heidelberg, Germany; 9 Division of Preventive Oncology, German Cancer Research Center (DKFZ) and National Center for Tumor Diseases (NCT), Heidelberg, Germany; 10 German Cancer Consortium (DKTK), German Cancer Research Center (DKFZ), Heidelberg, Germany; 11 University of Utrecht, Utrecht, The Netherlands; 12 Department of Digestive Tract Diseases, Medical University of Lodz, Lodz, Poland; 13 Division of Gastroenterology and Research Laboratory, Fondazione IRCCS “Casa Sollievo Della Sofferenza” Hospital, San Giovanni Rotondo, Italy; 14 Oncology of Massa Carrara, Oncological Department, Azienda USL Toscana Nord Ovest, Carrara, Italy; 15 Department of Oncology, Faculty of Medicine and Dentistry, Palacký University, Olomouc, Czech Republic; 16 Medical Faculty Heidelberg, Heidelberg University, Heidelberg, Germany; 17 Gastroenterology Department and Institute for Digestive Research, Lithuanian University of Health Sciences, Kaunas, Lithuania; 18 Institute for Translational Medicine, Medical School, University of Pécs, Pécs, Hungary; 19 Centre for Translational Medicine, Semmelweis University, Budapest, Hungary; 20 Division of Pancreatic Diseases, Heart and Vascular Center, Semmelweis University, Budapest, Hungary; 21 János Szentágothai Research Center, University of Pécs, Pécs, Hungary; 22 Surgery Clinic Third Faculty of Medicine, Charles University, Prague, Czech Republic; 23 Network Aging Research (NAR), Heidelberg University, Heidelberg, Germany; 24 Department of Molecular Biology of Cancer, Institute of Experimental Medicine of the Czech Academy of Sciences, Prague, Czech Republic; 25 First Faculty of Medicine, Institute of Biology and Medical Genetics, Charles University, Prague, Czech Republic; 26 Biomedical Center, Faculty of Medicine in Pilsen, Charles University, Pilsen, Czech Republic; 27 Department of General Surgery, University of Heidelberg, Heidelberg, Germany; 28 General Surgery, Department of Translational Research and New Technologies in Medicine and Surgery, University of Pisa, Pisa, Italy; 29 ARC-NET, Centre for Applied Research on Cancer, University and Hospital Trust of Verona, Verona, Italy; 30 Department DIMED-Laboratory Medicine, University of Padova, Padua, Italy; 31 Blood Transfusion Service, Azienda Ospedaliero-Universitaria Meyer, Florence, Italy; 32 Potenza Medical County Association, Potenza, Italy; 33 Division of General and Transplant Surgery, Pisa University Hospital, Pisa, Italy; 34 Department of Medical Oncology, Amsterdam UMC Location University of Amsterdam, Meibergdreef 9, Amsterdam, The Netherlands; 35 Cancer Center Amsterdam, Imaging and Biomarkers, Amsterdam, The Netherlands; 36 Centre for Translational Medicine, Department of Medicine, University of Szeged, Szeged, Hungary; 37 Center for Translational Medicine, Semmelweis University, Budapest, Hungary; 38 Department of Biomedical Sciences, Humanitas University, Milan, Italy; 39 Pancreatic Surgery Unit, IRCCS Humanitas Research Hospital, Milan, Italy; 40 Laboratory for Applied Science and Technology in Health, Carlos Chagas Institute, Oswaldo Cruz Foundation (Fiocruz), Curitiba, Brazil; 41 Department of Surgery, Erasmus MC University Medical Center, Rotterdam, The Netherlands; 42 Gastroenterology and Gastrointestinal Endoscopy Unit, Vita-Salute San Raffaele University, IRCCS San Raffaele Scientific Institute, Milan, Italy; 43 Genomic Epidemiology Group, German Cancer Research Center (DKFZ), Heidelberg, Germany

**Keywords:** Cancer genetics, Pancreatic cancer, Predictive markers, Risk factors

## Abstract

The incidence of pancreatic ductal adenocarcinoma (PDAC) is different among males and females. This disparity cannot be fully explained by the difference in terms of exposure to known risk factors; therefore, the lower incidence in women could be attributed to sex-specific hormones. A two-phase association study was conducted in 12,387 female subjects (5436 PDAC cases and 6951 controls) to assess the effect on risk of developing PDAC of single nucleotide polymorphisms (SNPs) in 208 genes involved in oestrogen and pregnenolone biosynthesis and oestrogen-mediated signalling. In the discovery phase 14 polymorphisms showed a statistically significant association (P < 0.05). In the replication none of the findings were validated. In addition, a gene-based analysis was performed on the 208 selected genes. Four genes (*NR5A2*, *MED1*, *NCOA2* and *RUNX1*) were associated with PDAC risk, but only *NR5A2* showed an association (P = 4.08 × 10^−5^) below the Bonferroni-corrected threshold of statistical significance. In conclusion, despite differences in incidence between males and females, our study did not identify an effect of common polymorphisms in the oestrogen and pregnenolone pathways in relation to PDAC susceptibility. However, we validated the previously reported association between *NR5A2* gene variants and PDAC risk.

## Introduction

Pancreatic ductal adenocarcinoma (PDAC), the most common form of pancreatic cancer, will become the 2nd leading cause of cancer-related mortality by 2030^1^. PDAC is a relatively rare disease with a reported incidence which is slightly higher in men compared to women (5.7/100,000 new case every year worldwide in males, while 4.1/100,000 new case every year worldwide in females)^2,3^. The known disparity in terms of exposure to known risk factors, such as smoking and heavy alcohol consumption can only partially explain this difference^4^. Therefore, it has been hypothesized that hormonal factors might account for this unbalance.

Sex steroid hormones (oestrogens, progesterone and androgens) exert their effect by binding to specific receptors, the function of which is tissue- and cell type-specific. Two known oestrogens receptors (ESR; estrogen receptor 1 (Erα) and estrogen receptor 2 (Erβ)) are expressed in the normal exocrine pancreas and in animal models the growth of pre-neoplastic pancreatic lesions or pancreatic carcinoma is inhibited by oestrogens^5^. Additionally, hormone replacement therapy (HRT) reduces insulin level, that is a risk factor for PDAC^6^. However, whether female sex steroid hormones have a role in PDAC onset, is still under debate^7^.

Previous studies on the association between reproductive factors and exposure to sex hormones and the risk of developing PDAC have shown heterogeneous results. For example older age at menarche, use of oral contraceptives (OC), and the use of hormone replacement therapy (HRT) have been reported to be associated with increased^8–10^, but also with decreased risk^10,11^ of developing PDAC. Furthermore several studies have also reported a null association^12–14^.

There is strong evidence of the role of single nucleotide polymorphisms (SNPs), on PDAC susceptibility^15–29^. Additionally, SNPs in oestrogen-related genes are associated with the susceptibility of several cancer types, such as breast, gastric, lung and prostate^30–35^. Therefore, it is surprising that none of the previous reports on female reproductive factors have considered the possible role of polymorphisms in genes involved in female hormone activity as factors contributing to PDAC susceptibility. We considered 36,454 SNPs in 208 genes involved in pregnenolone biosynthesis, oestrogen biosynthesis and ESR-mediated signaling and evaluated their role in PDAC risk. The study was carried out in 5436 female PDAC patients and 6951 women without PDAC in the context of the Pancreatic Cancer Cohort Consortium (PanScan) I, II, III, Pancreatic Cancer Case–Control Consortium (PanC4) and PANcreatic Disease ReseArch (PANDoRA) studies.

## Results

To investigate the role of polymorphic variants in oestrogen-related genes in PDAC, we utilized a three-phase (identification, discovery, validation) approach.

As a first step, the identification phase, 208 genes involved in oestrogen and pregnenolone pathways were selected using the reactome database (https://reactome.org/). All common SNPs (minor allele frequency > 0.01) in each gene region were identified. To include regulatory variants, 1000 base pairs were added before the first exon and after the last exon of each gene. A total of 23,569 SNPs, with a minor allele frequency (MAF) > 0.01, were present in the 208 genes. The list of SNPs was thinned down using linkage disequilibrium (LD), utilizing a threshold of r^2^ ≥ 0.80. This step was carried out to obtain a list of 12,885 independent SNPs (more details on the procedure are given in the material and methods section of the manuscript). This final list of 12,885 SNPs was analyzed in the discovery phase of the study that consisted of 3986 female PDAC cases and 3218 female controls belonging to PanScan I-III and PanC4 studies. To carry out this analysis the raw genotyping data were downloaded from the database of Genotypes and Phenotypes (dbGaP) (https://www.ncbi.nlm.nih.gov/gap/). In the discovery phase we observed 9 genes (*NR5A2, NRAS*, *ERBB4*, *PIK3CA, HSD17B11, EGFR NCOA2*, *PTGES3* and *POLR2A*) with at least one polymorphic variant (14 in total) that showed a statistically significant association with PDAC risk (p < 0.05). The results of the discovery phase are reported in Supplementary Table S1. Among these 9 genes, *NR5A2* was already reported as a PDAC risk locus, in a genome-wide association study (GWAS) on PDAC susceptibility^16^ that was carried out in PanScan II and validated in the context of the PANDoRA consortium^36^. In the validation phase, 1 SNP in each gene was analyzed in PANDoRA. None of the SNPs analyzed in PANDoRA showed allelic and genotypic frequency that deviated from Hardy–Weinberg equilibrium (HWE) and the genotyping concordance between duplicates was higher than 99%. In PANDoRA none of the SNPs showed a statistically significant association with PDAC susceptibility. However, *NR5A2-*rs2816945 showed a borderline association (OR = 1.16 (95% CI 0.98–1.38), P = 0.091). In the meta-analysis of the two phases, four SNPs showed a statistically significant association. More in detail, the G allele of the *NR5A2-*rs2816945 SNP and the T allele of the *ERBB4*-rs11904566 SNP were associated with increased PDAC risk (P = 9.57 × 10^−5^ and P = 1.16 × 10^−2^ respectively), while the A allele of the *EGFR-*rs138154852 SNP and the T allele of the *POLR2A-*rs*8753* SNP were associated with decreased PDAC risk (P = 5.93 × 10^−4^ and P = 5.25 × 10^−3^). However, none of the SNPs showed a P-value lower than the threshold of significance adopted considering a correction for multiple testing (P = 3.88 × 10^−6^). All the results are shown in Table 1.

**Table 1:** Results of the analysis of the nine candidate SNPs selected after the discovery phase of the study.

Chr	Gene	SNP	Position	Alleles (M/m)^a^	MAF (cases/controls)	Phase	OR (95% CI)	P-value
1	*NR5A2*	rs2816945	199,992,365	C/G	0.257/0.228	PanScan + PanC4	**1.17 (1.08–1.26)**	**8.39 × 10**^**−5**^
					0.218/0.219	PANDoRA	1.16 (0.98–1.38)	0.091
					0.248/0.225	Meta-analysis	**1.15 (1.07–1.23)**	**9.57 × 10**^**−5**^
1	*NRAS*	rs8453*	115,259,599	G/T	0.156/0.141	PanScan + PanC4	**1.15 (1.05–1.27)**	**2.64 × 10**^**−3**^
					0.138/0.144	PANDoRA	0.98 (0.81–1.19)	0.86
					0.151/0.143	Meta-analysis	1.12 (0.89–1.42)	0.332
2	*ERBB4*	rs11904566	212,354,011	A/G	0.028/0.020	PanScan + PanC4	**1.41 (1.13–1.76)**	**2.53 × 10**^**−3**^
					0.036/0.033	PANDoRA	1.03 (0.78–1.38)	0.788
					0.030/0.027	Meta-analysis	**1.25 (1.05–1.49)**	**1.16 × 10**^**−2**^
3	*PIK3CA*	rs61796467*	178,900,596	G/A	0.074/0.059	PanScan + PanC4	**1.26 (1.10–1.44)**	**1.03 × 10**^**−3**^
					0.083/0.077	PANDoRA	0.98 (0.81–1.19)	0.86
					0.076/0.069	Meta-analysis	1.12 (0.89–1.42)	0.332
4	*HSD17B11*	rs116113712*	88,295,123	G/A	0.017/0.011	PanScan + PanC4	**1.60 (1.17–2.10)**	**1.43 × 10**^**−3**^
					0.005/0.010	PANDoRA	0.78 (0.40–1.49)	0.45
					0.014/0.010	Meta-analysis	1.20 (0.62–2.32)	0.596
7	*EGFR*	rs138154852	55,125,950	G/A	0.054/0.068	PanScan + PanC4	**0.78 (0.68–0.90)**	**4.98 × 10**^**−4**^
					0.070/0.076	PANDoRA	0.90 (0.70–1.15)	0.381
					0.057/0.071	Meta-analysis	**0.81 (0.71–0.91)**	**5.93 × 10**^−**4**^
8	*NCOA2*	rs113654977	71,164,275	T/C	0.031/0.040	PanScan + PanC4	**0.72 (0.60–0.87)**	**4.62 × 10**^**−4**^
					0.043/0.037	PANDoRA	1.17 (0.95–1.51)	0.255
					0.034/0.039	Meta-analysis	0.94 (0.58–1.45)	0.697
12	*PTGES3*	rs2950390*	57,055,291	C/T	0.326/0.346	PanScan + PanC4	**0.91 (0.85–0.97)**	**6.28 × 10**^**−3**^
					0.337/0.339	PANDoRA	1.05 (0.94–1.17)	0.379
					0.329/0.342	Meta-analysis	0.91 (0.85–1.11)	0.656
17	*POLR2A*	rs8753	7,417,640	C/T	0.015/0.022	PanScan + PanC4	**0.66 (0.51–0.85)**	**1.20 × 10**^**−3**^
					0.016/0.022	PANDoRA	0.75 (0.51–1.10)	0.140
					0.015/0.022	Meta-analysis	**0.69 (0.55–0.85)**	**5.25 × 10**^**−3**^

All analyses of PanScan and PanC4 data were adjusted by age, and the first 8 principal components. Analysis of PANDoRA data were adjusted for age and country of origin. The meta-analysis was performed applying the fixed-effects model, or random-effects model for SNPs showing heterogeneity. Statistically significant results (P < 0.05) are in bold.

^a^M stands for major allele, m stands for minor allele.

*Shows SNPs with heterogeneity.

For the 9 SNPs that were analyzed in the validation phase we also performed an analysis conducted in males only and the results show a statistically significant association for *NR5A2-*rs2816945 and no association for any of the other SNPs, confirming what observed in women and what already reported in the GWAS (Supplementary Table S2).

To further explore the associations between the genetic variability of the 208 estrogen-related genes in female PDAC patients, a gene-based analysis was also performed. The results, that consider the cumulative effect of all the SNPs belonging to the same gene, showed that *NR5A2* (P = 4.08 × 10^−5^), *MED1* (P = 3.14 × 10^−3^), *NCOA2* (P = 6.38 × 10^−3^) and *RUNX1* (P = 9.01 × 10^−3^), had a statistically significant association with PDAC risk. However, with the exception of *NR5A2*, none of these findings met the criteria for statistical significance after correction for multiple testing. The results of these analyses are reported in Table 2 (genes with a P-value for association < 0.05) and in Supplementary Table S3 (all genes).

**Table 2:** Significant results of the gene-based analysis.

Gene	Chr	N° SNPs	P_Multi_	P_SNPWiseMean_	P_SNPWiseTop1_	Pathways
*NR5A2*	1	502	4.08 × 10^−5^	2.51 × 10^−3^	2.19 × 10^−5^	ESR-mediated signalling
*MED1*	17	90	3.14 × 10^−3^	2.62 × 10^−3^	3.45 × 10^−2^	ESR-mediated signalling
*NCOA2*	8	439	6.38 × 10^−3^	2.73 × 10^−2^	9.71 × 10^−3^	ESR-mediated signalling
*RUNX1*	21	574	9.01 × 10^−3^	2.40 × 10^−2^	2.14 × 10^−2^	ESR-mediated signalling

The three models used are: (1) SNP-wise Mean, (2) SNP-wise Top 1 and (3) Multi model. The two SNP-wise models examine the individual SNPs present in the gene and subsequently combine the resulting P-values of the SNPs into a gene test statistic, while the multi model runs the basic models (SNP-wise) and combines the resulting P-values into an aggregated P-value for the gene. *NR5A2 is* significant after the Bonferroni correction (P < 0.05/208 = 2.40 × 10^−4^).

^a^The pathways was identified using the reactome website.

Finally, a pathway-based analysis, combining together all SNPs belonging to the same pathway, was also performed. The analysis did not show any statistically significant association (Supplementary Table S4).

## Discussion

The difference in the reported PDAC incidence between males and females has been usually explained by the different exposure of the two sexes to environmental and lifestyle factors, such as pollution, smoking and alcohol consumption^7^. However, several reports have suggested that the difference could be at least partially explained other factors such as oestrogen exposure^37,38^. Additionally, SNPs in oestrogen-related pathways have been associated with increased risk of developing several cancer types, such as breast, ovarian, prostate and lung^31,39–43^. For this reason, we have identified 12,885 tagging SNPs (tSNPs) in 208 genes belonging to the pregnenolone and oestrogen biosynthesis and ESR-mediated signaling pathways to test whether the genetic variability of these genes is associated with PDAC risk in females. We analyzed 13,371 women (5783 cases and 7588 controls).

Despite several signals in the discovery phase of the study, none of the selected SNPs showed a statistically significant association in the validation phase that consisted of 1450 female PDAC cases and 1128 controls belonging to the PANDoRA consortium. Even though not statistically significant, *NR5A2-*rs2816945 showed a trend (p = 0.091) with the G allele associated with increased risk. Polymorphic variants belonging to *NR5A2* have been already reported to be associated with in PDAC risk^16,27^. A polymorphic variant of the *NR5A2,* rs2821357, that is in LD with rs2816945 (r^2^ = 0.57, D′ = 0.99, in 1000 Genomes Europeans) is associated with low density cholesterol levels^44^. This is intriguing since estrogens, in the liver, decrease the total amount of LDL and increase the amount of HDL in the body and LDL is a suggested PDAC risk factor. Therefore, it can be hypothesized that the effect of *NR5A2-*rs2816945 on PDAC susceptibility might be mediated by its effect on cholesterol level. The gene-based analysis confirmed the association of *NR5A2* as a PDAC susceptibility gene. *NR5A2* belongs to the fushi tarazu factor-1 subfamily of orphan nuclear receptors and plays an essential role in a variety of biological processes that include endodermal development, cholesterol homeostasis, bile acid synthesis and steroidogenesis. The reactome database, that was used to select the genes of this study, includes NR5A2 in the ESR-mediated signaling pathway. In adult mammals, this gene is mainly expressed in the exocrine pancreas, ovary, liver and intestine^40^. In the pancreas, *NR5A2* cooperates with the pancreas-specific transcription factor 1 (*PTF1*) to maintain the secretory functions of acinar cells by regulating the expression of specific acinar genes. In vitro, it was observed that the loss of *NR5A2* leads to the downregulation of terminal acinar differentiation elements and to an increased chance to undergo acinar-to-ductal metaplasia (ADM)^42^. It has been suggested that loss of *NR5A2* expression represents a first step in the development of PDAC because provides a permissive environment for *KRAS* driven ADM and pancreatic intraepithelial neoplasia (PanIN) development^41,42^. Alongside *NR5A2*, the other three genes that showed a nominal (P < 0.05) association with PDAC risk (*MED1*, *NCOA2* and *RUNX1*) are all transcriptional regulators that are expressed in the pancreas and in many other tissues and have a broad range of functions, such as hematopoiesis, adipogenesis and lipid metabolism^45,46^. It is, therefore, difficult to establish a functional link between the genetic variability of the three genes and PDAC.

Other studies have explored the possible involvement of estrogen-related SNPs and risk of developing gastrointestinal cancers. For example, the study conducted by Park and colleagues, shows that the G allele of the *ESR1*-rs1801132 SNP was associated with increased risk of developing bile duct cancer (OR = 1.70, 95% CI 1.10–2.80, P = 0.07) compared with C allele.^47^. Another example is the study conducted by Lin et al. that investigated colorectal cancer risk in women only. In that study the authors report three SNPs, rs10046 in *CYP19A1*, rs2911422 and rs2042429 in *HSD17B2* genes, that were marginally associated with colorectal cancer risk.^48^.

Clear strengths of this study are the large sample size, the novelty of the focused analysis on women only for oestrogen-related genes and using a study design consisting of discovery and validation phases to avoid reporting false positives. A possible limitation consists in the fact that we have analyzed only relatively common SNPs (MAF > 0.01) with a low penetrance and therefore we could not exclude that rare variants in the selected genes might instead influence PDAC risk. Another possible limitation is that we used only the reactome database to select the genes of interest, with the consequence that we could have identified only a part of estrogen-related genes, since the overlap between different databases (e.g. Kyoto Encyclopedia of Genes and Genomes (KEGG) or NCI Pathway Interaction Database) is only partial. However, it is highly unlikely that we missed genes that are central to the pathways of our interest. Finally, data on exposome and gynecological/reproductive factors were not available either in PanScan and PanC4 or in PANDoRA. In conclusion, we have replicated a previously reported association in the *NR5A2* gene considering only women with PDAC and have not identified novel associations, suggesting that common SNPs in oestrogen-related genes do not play a major role in PDAC susceptibility.

## Materials and methods

**Figure 1:**
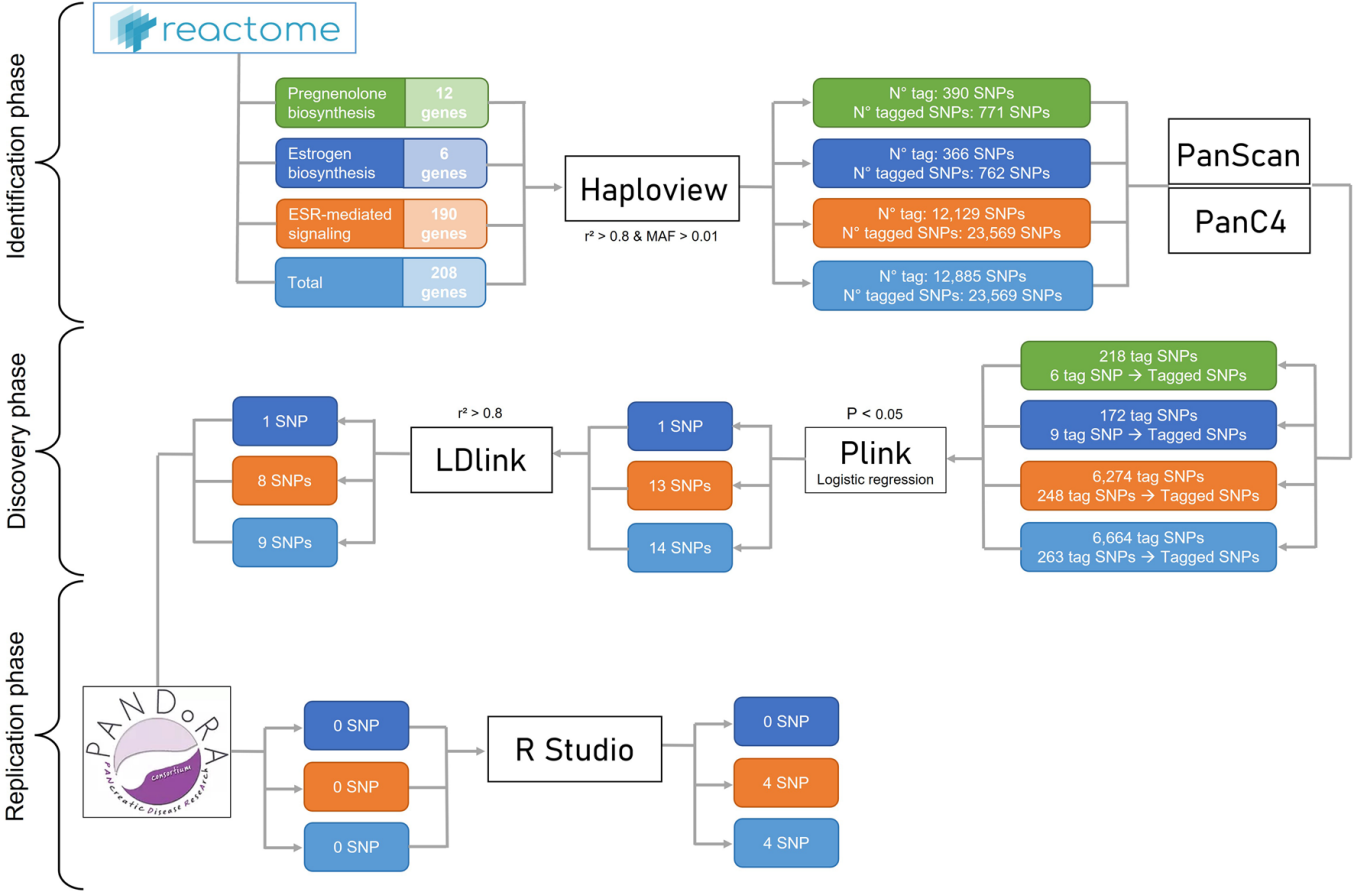
Workflow of the study. Flowchart of the three phases (identification, discovery, and validation) of the study. The colors of the boxes identify the different pathways analyzed: green for the pregnenolone biosynthesis, dark blue for the oestrogen biosynthesis, orange for the oestrogen receptor-mediated signaling, and light blue identifies all the previous three.

This study was carried out using three phases, identification, discovery, and validation. Figure 1 shows the workflow of the study.

### Identification phase

In the identification phase, the reactome (https://reactome.org/) database was used to select genes in three oestrogen and pregnenolone related pathways, namely the pregnenolone biosynthesis (number of genes = 12); the oestrogen biosynthesis (number of genes = 6) and the ESR-mediated signaling (number of genes = 190) for a total of 208 genes^49^. Tagging SNPs (tSNPs) were identified in each gene region, defined as the region between the beginning of the first known exon and the end of the last known exon, according to 1000 Genomes, with the addition of 1000 bp on each end of the gene. We performed pairwise tagging using genotype data from Ensembl v80 GRCh37, with the use of the Tagger program within Haploview (http://www.broad.mit.edu/mpg/haploview/; http://www.broad.mit.edu/mpg/tagger/). The following criteria were used: minor allele frequency (MAF) > 0.01 in 1000 Genomes subjects of European descent, r^2^ ≥ 0.8. A total of 23,569 SNPs were captured with 12,885 tagging SNPs. Supplementary Table S1 shows all the genes, the number of SNPs and tSNPs, divided by gene, analyzed in the study.

### Discovery phase

All SNPs identified were analysed using the genotypes of the PanScan I, II, III and PanC4 GWASs. The genotypes of 9563 PDAC cases and 8073 controls were downloaded from the database of Genotypes and Phenotypes (dbGaP; study accession nos. phs000206.v5.p3 and phs000648.v1.p1; project reference no. 12644). Genotyping procedures, quality control and data collection details of these studies have been previously described in the original publications^15,23,24,50^. After downloading the datasets, we carried out quality controls (QCs) and imputation. The QCs were performed prior to the imputation and included: removal of individuals with gender mismatches, call rate < 0.98, minimal or excessive heterozygosity (> 3 SDs from the mean) or cryptic relatedness (PI_HAT > 0.2) and exclusion of SNPs with minor allele frequency (MAF) < 0.01, call rate < 0.98 or evidence for violations of Hardy–Weinberg equilibrium (P < 1 × 10^−6^). The genotypes were phased using SHAPEIT v2 software and the imputation was performed, separately for each dataset, using the Michigan Imputation Server (https://imputationserver.sph.umich.edu), and the Haplotype Reference Consortium (HRC) as reference, and merged using PLINK 2.0 software^51^. Afterwards the SNPs with completion rate and call-rate < 98%, a minor allele frequency (MAF) < 0.01, evidence for violations of Hardy–Weinberg equilibrium (P < 1 × 10^−5^) and low-quality imputation score (INFO score < 0.7) were discarded, leaving 7,509,345 SNPs in the final dataset. Principal component analysis was carried out to exclude individuals not clustering within Europeans. All male subjects were then removed from the dataset, leaving a total of 7207 women (3986 PDAC cases and 3218 controls). A logistic regression analysis adjusted for sex, age and the top eight principal components was used to test the association between the SNPs and PDAC risk.

### Validation phase

The significant SNPs identified in the discovery phase were genotyped in 1450 PDAC female cases and 1128 female controls belonging to the pancreatic disease research (PANDoRA) consortium. The PANDoRA consortium has been extensively described elsewhere^52^. Briefly, it consist of a multicentric study conducted in 10 European countries (Italy, Greece, Germany, Netherlands, Denmark, Czech Republic, Hungary, Poland, Lithuania and United Kingdom), and Brazil. Cases had a confirmed diagnosis of PDAC and data on age at diagnosis, sex and country of origin was retrospectively collected for each patient. Controls were selected from blood donors, the general population and hospitalised subjects without oncological diseases. In addition to PANDoRA subjects, the genotypes of 55 British and 38 Dutch controls from the European Prospective Investigation into Cancer and Nutrition (EPIC), a prospective cohort study with 519,978 participants (aged 35–70 years) from ten European countries^53^, and 2,512 German controls from Epidemiologische Studie zu Chancen der Verhütung, Früherkennung und optimierten THerapie chronischer ERkrankungen in der älteren Bevölkerung (ESTHER), a cohort study that includes 9,961 German people aged between 50 and 74 years^54^, were included. Genotyping was conducted using Taqman assays (ThermoFisher Applied Biosystems, Waltham MA, USA) in 384 well plates, using 8% of duplicated samples to ensure quality control of the laboratory procedure. In each plate an approximately equal number of cases and controls were used. Genotyping calls were made using QuantStudioTM 5 Real-Time PCR system (Thermofisher, USA) and QuantStudio software. Hardy–Weinberg equilibrium was checked for all SNPs in the controls. The association analysis was performed with logistic regression adjusting for sex, age (at diagnosis for cases and at recruitment for controls) and country of origin (PANDoRA lacks GWAS data, therefore principal component analysis cannot be performed).

Finally, a fixed effect meta-analysis between the results of the two phases was conducted in the 12,387 individuals included in the two study phases using R software package. The p-value threshold for statistical significance for the individual SNPs was set at 0.05/12,885 = 3.88 × 10^−6^ considering the number of independent SNPs (r^2^ < 0.80) analyzed in the discovery phase.

### Gene based analysis

Additionally, a gene-based and pathways-based analysis were also conducted using the Multi-marker Analysis of GenoMic Annotation (MAGMA) software^55^. These analyses were restricted to PanScan I-III and PanC4, since for PANDoRA GWAs data are not available. The p value threshold to consider an association statistically significant for the gene based analysis was 0.05/208 = 2.40 × 10^−4^.

### Ethics statement

Each participant in the PanScan and PanC4 studies obtained approval from the responsible institutional review board (IRB) and IRB certification permitting data sharing in accordance with the NIH Policy for sharing of Data Obtained in NIH-Supported or NIH-Conducted Genome Wide Association Studies. The PANDoRA study protocol was approved by the Ethics Commission of the Medical Faculty of the University of Heidelberg. In accordance with the Declaration of Helsinki, written informed consent was obtained from each participant.

## Supplementary Information

Supplementary Tables.

## Data Availability

The PanScan and PanC4 genotyping data are available from the database of Genotypes and Phenotypes (dbGaP, study accession numbers phs000206.v5.p3 and phs000648.v1.p1). The PANDoRA primary data for this work will be made available to researchers who submit a reasonable request to the corresponding author, conditional to approval by the PANDoRA Steering Committee and Ethics Commission of the Medical Faculty of the University of Heidelberg. Data will be stripped from all information allowing identification of study participants.
