# Supplementary material for: Common variability in oestrogen-related genes and pancreatic ductal adenocarcinoma risk in women
Source: Sci Rep. 2022 Oct 27;12:18100. doi: 10.1038/s41598-022-22973-9 (PMC9613634; doi:10.1038/s41598-022-22973-9)
Supplement: Supplementary file 1 — Supplementary Tables. [file 41598_2022_22973_MOESM1_ESM.docx]

**Supplementary table S1.** Results of the discovery phase.

| **Pathway** | **Gene** | **Chr** | **SNP** | **Alleles (M/m)^a^** | **OR (95% CI)** | **P-value** |
| --- | --- | --- | --- | --- | --- | --- |
| ESR-mediated signaling | *NR5A2* | 1 | rs61486545 | G/A | 0.82 (0.76-0.89) | 8.02×10⁻⁷ |
| ESR-mediated signaling | *NR5A2* | 1 | rs3790843 | C/T | 0.83 (0.77-0.90) | 2.17×10⁻⁶ |
| ESR-mediated signaling | *NR5A2* | 1 | rs2816945 | C/G | 1.17 (1.08-1.26) | 8.39×10⁻⁵ |
| ESR-mediated signaling | *NR5A2* | 1 | rs2737657 | G/A | 1.17 (1.08-1.27) | 8.68×10⁻⁵ |
| ESR-mediated signaling | *NR5A2* | 1 | rs2821371 | G/A | 1.14 (1.07-1.23) | 1.75×10⁻⁴ |
| ESR-mediated signaling | *NR5A2* | 1 | rs2977425 | G/A | 1.16 (1.06-1.28) | 2.45×10⁻³ |
| ESR-mediated signaling | *NRAS* | 1 | rs8453 | G/T | 1.15 (1.05-1.27) | 2.64×10⁻³ |
| ESR-mediated signaling | *ERBB4* | 2 | rs11904566 | A/G | 1.41 (1.13-1.76) | 2.53×10⁻³ |
| ESR-mediated signaling | *PIK3CA* | 3 | rs61796467 | G/A | 1.26 (1.10-1.44) | 1.03×10⁻³ |
| Estrogen biosynthesis | *HSD17B11* | 4 | rs116113712 | G/A | 1.60 (1.17-2.10) | 1.43×10⁻³ |
| ESR-mediated signaling | *EGFR* | 7 | rs138154852 | G/A | 0.78 (0.68-0.90) | 4.98×10⁻⁴ |
| ESR-mediated signaling | *NCOA2* | 8 | rs113654977 | T/C | 0.72 (0.60-0.87) | 4.62×10⁻⁴ |
| ESR-mediated signaling | *PTGES3* | 12 | rs2950390 | C/T | 0.91 (0.85-0.97) | 6.28×10⁻³ |
| ESR-mediated signaling | *POLR2A* | 17 | rs8753 | C/T | 0.66 (0.51-0.85) | 1.20×10⁻³ |

All analyses of PanScan and PanC4 data were adjusted by age and the first 8 principal components.

^a^ M stands for major allele, m stands for minor allele.

**Supplementary table S2.** Results of the analysis of the nine candidate SNPs selected after the discovery phase of the study in male subjects.

| **Chr** | **Gene** | **SNP** | **Position** | **Alleles (M/m)** | **MAF (cases/controls)** | **Phase** | **OR (95% CI)** | **P-value** |
| --- | --- | --- | --- | --- | --- | --- | --- | --- |
| 1 | *NR5A2* | rs2816945 | 199992365 | C/G | 0.261/0.222 | PanScan+PanC4 | **1.24 (1.15-1.33)** | **5.45×10⁻⁹** |
|  |  |  |  |  | 0.217/0.200 | PANDoRA | 1.12 (0.99-1.27) | 0.065 |
|  |  |  |  |  | 0.251/0.215 | Meta-analysis | **1.21 (1.13-1.29)** | **3.48×10⁻⁹** |
| 1 | *NRAS* | rs8453 | 115259599 | G/T | 0.147/0.149 | PanScan+PanC4 | 1.00 (0.92-1.09) | 0.974 |
|  |  |  |  |  | 0.136/0.144 | PANDoRA | 0.99 (0.80-1.22) | 0.902 |
|  |  |  |  |  | 0.144/0.148 | Meta-analysis | 1.00 (0.92-1.08) | 0.972 |
| 2 | *ERBB4* | rs11904566 | 212354011 | A/G | 0.025/0.023 | PanScan+PanC4 | 1.09 (0.90-1.33) | 0.375 |
|  |  |  |  |  | 0.033/0.036 | PANDoRA | 0.93 (0.70-1.22) | 0.586 |
|  |  |  |  |  | 0.027/0.027 | Meta-analysis | 1.02 (0.86-1.23) | 0.7611 |
| 3 | *PIK3CA* | rs61796467 | 178900596 | G/A | 0.067/0.067 | PanScan+PanC4 | 0.95 (0.84-1.07) | 0.409 |
|  |  |  |  |  | 0.086/0.082 | PANDoRA | 1.00 (0.84-1.21) | 0.939 |
|  |  |  |  |  | 0.072/0.073 | Meta-analysis | 0.97 (0.87-1.07) | 0.497 |
| 4 | *HSD17B11* | rs116113712 | 88295123 | G/A | 0.015/0.013 | PanScan+PanC4 | 1.20 (0.92-1.55) | 0.172 |
|  |  |  |  |  | 0.009/0.010 | PANDoRA | 0.91 (0.55-1.52) | 0.726 |
|  |  |  |  |  | 0.013/0.012 | Meta-analysis | 1.11 (0.83-1.48) | 0.466 |
| 7 | *EGFR* | rs138154852 | 55125950 | G/A | 0.063/0.060 | PanScan+PanC4 | 1.07 (0.94-1.22) | 0.321 |
|  |  |  |  |  | 0.074/0.074 | PANDoRA | 1.00 (0.82-1.20) | 0.953 |
|  |  |  |  |  | 0.064/0.064 | Meta-analysis | 1.05 (0.94-1.17) | 0.401 |
| 8 | *NCOA2* | rs113654977 | 71164275 | T/C | 0.033/0.036 | PanScan+PanC4 | 0.89 (0.76-1.06) | 0.186 |
|  |  |  |  |  | 0.041/0.030 | PANDoRA | 1.07 (0.77-1.50) | 0.686 |
|  |  |  |  |  | 0.035/0.035 | Meta-analysis | 0.94 (0.77-1.13) | 0.485 |
| 12 | *PTGES3* | rs2950390 | 57055291 | C/T | 0.336/0.337 | PanScan+PanC4 | 1.00 (0.93-1.06) | 0.884 |
|  |  |  |  |  | 0.325/0.328 | PANDoRA | 0.96 (0.86-1.07) | 0.426 |
|  |  |  |  |  | 0.333/0.334 | Meta-analysis | 0.99 (0.93-1.05) | 0.705 |
| 17 | *POLR2A* | rs8753 | 7417640 | C/T | 0.016/ 0.019 | PanScan+PanC4 | 0.85 (0.67-1.07) | 0.164 |
|  |  |  |  |  | 0.017/0.019 | PANDoRA | 1.30 (0.81-2.08) | 0.274 |
|  |  |  |  |  | 0.016/0.019 | Meta-analysis | 1.00 (0.67-1.48) | 0.985 |

All analyses of PanScan and PanC4 data were adjusted by age and the first 8 principal components. Analysis of PANDoRA data were adjusted for age and country of origin. The meta-analysis was performed applying the fixed-effects model, or random-effects model for SNPs showing heterogeneity. Statistically significant results (P<0.05) are in bold.

^a^ M stands for major allele, m stands for minor allele.

* Shows SNPs with heterogeneity

**Supplementary table S3.** Results of gene-based analysis performed on the PanScan I, II, III and PanC4 population

| **Gene** | **Chr** | **N° SNPs** | **P_Multi_** | **P_SNPWiseMean_** | **P_SNPWiseTop1_** | **Pathway** |
| --- | --- | --- | --- | --- | --- | --- |
| *NR5A2* | 1 | 502 | 4.08×10^-5^ | 2.51×10^-3^ | 2.19×10^-5^ | ESR-mediated signaling |
| *NRAS* | 1 | 27 | 6.66×10^-2^ | 0.178 | 3.49×10^-2^ | ESR-mediated signaling |
| *AKT3* | 1 | 419 | 8.88×10^-2^ | 0.141 | 7.57×10^-2^ | ESR-mediated signaling |
| *GNG5* | 1 | 62 | 0.109 | 0.118 | 0.116 | ESR-mediated signaling |
| *GNG12* | 1 | 315 | 0.139 | 0.149 | 0.154 | ESR-mediated signaling |
| *AGO4* | 1 | 56 | 0.153 | 6.56×10^-2^ | 0.373 | ESR-mediated signaling |
| *POU2F1* | 1 | 288 | 0.277 | 0.163 | 0.468 | ESR-mediated signaling |
| *PIK3R3* | 1 | 219 | 0.279 | 0.223 | 0.345 | ESR-mediated signaling |
| *JUN* | 1 | 20 | 0.367 | 0.273 | 0.475 | ESR-mediated signaling |
| *GNAI3* | 1 | 68 | 0.382 | 0.541 | 0.249 | ESR-mediated signaling |
| *HDAC1* | 1 | 20 | 0.414 | 0.452 | 0.354 | ESR-mediated signaling |
| *MOV10* | 1 | 69 | 0.432 | 0.503 | 0.334 | ESR-mediated signaling |
| *KDM1A* | 1 | 77 | 0.548 | 0.743 | 0.354 | ESR-mediated signaling |
| *HIST2H2AA3* | 1 | 6 | 0.592 | 0.561 | 0.615 | ESR-mediated signaling |
| *H2AZ1* | 1 | 26 | 0.660 | 0.561 | 0.708 | ESR-mediated signaling |
| *HIST3H2BB* | 1 | 16 | 0.719 | 0.713 | 0.654 | ESR-mediated signaling |
| *AGO1* | 1 | 61 | 0.725 | 0.843 | 0.541 | ESR-mediated signaling |
| *GNB1* | 1 | 108 | 0.749 | 0.751 | 0.696 | ESR-mediated signaling |
| *USF1* | 1 | 41 | 0.758 | 0.610 | 0.862 | ESR-mediated signaling |
| *GNG4* | 1 | 297 | 0.775 | 0.681 | 0.722 | ESR-mediated signaling |
| *AGO3* | 1 | 11 | 0.781 | 0.823 | 0.698 | ESR-mediated signaling |
| *H3-3A* | 1 | 23 | 0.799 | 0.782 | 0.723 | ESR-mediated signaling |
| *PRKCZ* | 1 | 268 | 0.845 | 0.817 | 0.797 | ESR-mediated signaling |
| *UHMK1* | 1 | 94 | 0.913 | 0.994 | 0.709 | ESR-mediated signaling |
| *SHC1* | 1 | 18 | 0.959 | 0.903 | 0.952 | ESR-mediated signaling |
| *HIST2H2BE* | 1 | 11 | 0.971 | 0.944 | 0.958 | ESR-mediated signaling |
| *HIST2H2AC* | 1 | 10 | 0.971 | 0.948 | 0.958 | ESR-mediated signaling |
| *XPO1* | 2 | 109 | 0.353 | 0.227 | 0.505 | ESR-mediated signaling |
| *GREB1* | 2 | 307 | 0.411 | 0.429 | 0.366 | ESR-mediated signaling |
| *TGFA* | 2 | 347 | 0.443 | 0.244 | 0.744 | ESR-mediated signaling |
| *ERBB4* | 2 | 3940 | 0.446 | 0.413 | 0.419 | ESR-mediated signaling |
| *CREB1* | 2 | 114 | 0.489 | 0.489 | 0.416 | ESR-mediated signaling |
| *NCOA1* | 2 | 589 | 0.533 | 0.476 | 0.527 | ESR-mediated signaling |
| *STRN* | 2 | 324 | 0.574 | 0.359 | 0.755 | ESR-mediated signaling |
| *ATF2* | 2 | 202 | 0.818 | 0.769 | 0.752 | ESR-mediated signaling |
| *POLR2D* | 2 | 63 | 0.910 | 0.856 | 0.862 | ESR-mediated signaling |
| *PIK3CA* | 3 | 204 | 3.53×10^-2^ | 7.62×10^-2^ | 2.91×10^-2^ | ESR-mediated signaling |
| *STAG1* | 3 | 569 | 9.48×10^-2^ | 0.130 | 9.30×10^-2^ | ESR-mediated signaling |
| *POLR2H* | 3 | 27 | 0.258 | 0.192 | 0.346 | ESR-mediated signaling |
| *GNAI2* | 3 | 47 | 0.279 | 0.245 | 0.315 | ESR-mediated signaling |
| *GNB4* | 3 | 98 | 0.361 | 0.261 | 0.474 | ESR-mediated signaling |
| *KCTD6* | 3 | 29 | 0.443 | 0.493 | 0.380 | ESR-mediated signaling |
| *KAT2B* | 3 | 471 | 0.708 | 0.635 | 0.708 | ESR-mediated signaling |
| *HSD17B11* | 4 | 192 | 5.82×10^-2^ | 0.112 | 4.76×10^-2^ | Oestrogen biosynthesis |
| *AREG* | 4 | 7 | 8.62×10^-2^ | 0.143 | 5.51×10^-2^ | ESR-mediated signaling |
| *EGF* | 4 | 198 | 0.133 | 0.167 | 0.128 | ESR-mediated signaling |
| *EPGN* | 4 | 31 | 0.230 | 0.398 | 0.137 | ESR-mediated signaling |
| *PPID* | 4 | 27 | 0.281 | 0.232 | 0.335 | ESR-mediated signaling |
| *POLR2B* | 4 | 174 | 0.373 | 0.353 | 0.365 | ESR-mediated signaling |
| *BTC* | 4 | 206 | 0.436 | 0.280 | 0.627 | ESR-mediated signaling |
| *EREG* | 4 | 112 | 0.816 | 0.700 | 0.792 | ESR-mediated signaling |
| *STARD4* | 5 | 27 | 0.349 | 0.325 | 0.353 | Pregnenolone biosynthesis |
| *HBEGF* | 5 | 44 | 0.368 | 0.478 | 0.263 | ESR-mediated signaling |
| *CXXC5* | 5 | 64 | 0.551 | 0.523 | 0.496 | ESR-mediated signaling |
| *CHD1* | 5 | 153 | 0.565 | 0.420 | 0.641 | ESR-mediated signaling |
| *PIK3R1* | 5 | 237 | 0.940 | 0.858 | 0.938 | ESR-mediated signaling |
| *HSP90AB1* | 6 | 50 | 5.24×10^-2^ | 6.59×10^-2^ | 5.93×10^-2^ | ESR-mediated signaling |
| *H2BC14* | 6 | 19 | 0.105 | 0.218 | 6.00×10^-2^ | ESR-mediated signaling |
| *H2AC14* | 6 | 16 | 0.106 | 0.284 | 4.43×10^-2^ | ESR-mediated signaling |
| *H2BC13* | 6 | 18 | 0.112 | 0.225 | 6.52×10^-2^ | ESR-mediated signaling |
| *H2BC15* | 6 | 17 | 0.220 | 0.469 | 0.106 | ESR-mediated signaling |
| *H2BC11* | 6 | 15 | 0.243 | 0.304 | 0.197 | ESR-mediated signaling |
| *H4C1* | 6 | 28 | 0.322 | 0.178 | 0.562 | ESR-mediated signaling |
| *FKBP5* | 6 | 287 | 0.389 | 0.214 | 0.625 | ESR-mediated signaling |
| *H3C1* | 6 | 26 | 0.391 | 0.242 | 0.596 | ESR-mediated signaling |
| *TBP* | 6 | 64 | 0.501 | 0.360 | 0.638 | ESR-mediated signaling |
| *H2BC12* | 6 | 24 | 0.523 | 0.828 | 0.294 | ESR-mediated signaling |
| *HIST1H2BB* | 6 | 30 | 0.525 | 0.398 | 0.627 | ESR-mediated signaling |
| *H2AC4* | 6 | 26 | 0.527 | 0.468 | 0.551 | ESR-mediated signaling |
| *ESR1* | 6 | 1188 | 0.579 | 0.827 | 0.337 | ESR-mediated signaling |
| *SRF* | 6 | 38 | 0.614 | 0.586 | 0.557 | ESR-mediated signaling |
| *HIST1H2BO* | 6 | 120 | 0.648 | 0.736 | 0.480 | ESR-mediated signaling |
| *FOXO3* | 6 | 171 | 0.678 | 0.543 | 0.745 | ESR-mediated signaling |
| *MYB* | 6 | 77 | 0.681 | 0.562 | 0.688 | ESR-mediated signaling |
| *H2BC1* | 6 | 43 | 0.718 | 0.918 | 0.482 | ESR-mediated signaling |
| *HIST1H2AC* | 6 | 68 | 0.806 | 0.692 | 0.812 | ESR-mediated signaling |
| *H2BC4* | 6 | 73 | 0.838 | 0.679 | 0.894 | ESR-mediated signaling |
| *HIST1H2BH* | 6 | 42 | 0.935 | 0.972 | 0.833 | ESR-mediated signaling |
| *H2AC7* | 6 | 57 | 0.940 | 0.875 | 0.921 | ESR-mediated signaling |
| *HIST1H2BD* | 6 | 34 | 0.955 | 0.962 | 0.897 | ESR-mediated signaling |
| *GNB2* | 7 | 23 | 2.19×10^-2^ | 1.20×10^-2^ | 6.75×10^-2^ | ESR-mediated signaling |
| *EGFR* | 7 | 558 | 0.149 | 0.415 | 6.07×10^-2^ | ESR-mediated signaling |
| *AKR1B1* | 7 | 62 | 0.171 | 0.432 | 7.32×10^-2^ | Pregnenolone biosynthesis |
| *AKR1B15* | 7 | 123 | 0.385 | 0.284 | 0.479 | Oestrogen biosynthesis |
| *GNAI1* | 7 | 213 | 0.433 | 0.753 | 0.223 | ESR-mediated signaling |
| *GNAT3* | 7 | 146 | 0.440 | 0.397 | 0.441 | ESR-mediated signaling |
| *POLR2J* | 7 | 41 | 0.488 | 0.293 | 0.740 | ESR-mediated signaling |
| *CAV2* | 7 | 71 | 0.687 | 0.814 | 0.524 | ESR-mediated signaling |
| *GNG11* | 7 | 38 | 0.704 | 0.554 | 0.796 | ESR-mediated signaling |
| *NOS3* | 7 | 51 | 0.714 | 0.553 | 0.805 | ESR-mediated signaling |
| *STARD3NL* | 7 | 184 | 0.778 | 0.654 | 0.817 | Pregnenolone biosynthesis |
| *GNGT1* | 7 | 53 | 0.807 | 0.735 | 0.797 | ESR-mediated signaling |
| *HSPB1* | 7 | 47 | 0.880 | 0.815 | 0.855 | ESR-mediated signaling |
| *CAV1* | 7 | 120 | 0.890 | 0.853 | 0.817 | ESR-mediated signaling |
| *H2AFV* | 7 | 70 | 0.958 | 0.909 | 0.961 | ESR-mediated signaling |
| *NCOA2* | 8 | 439 | 6.38×10^-3^ | 2.73×10^-2^ | 9.71×10^-3^ | ESR-mediated signaling |
| *RAD21* | 8 | 92 | 0.127 | 0.107 | 0.192 | ESR-mediated signaling |
| *MYC* | 8 | 35 | 0.234 | 0.168 | 0.338 | ESR-mediated signaling |
| *STAR* | 8 | 8 | 0.281 | 0.476 | 0.164 | Pregnenolone biosynthesis |
| *PTK2* | 8 | 468 | 0.285 | 0.177 | 0.454 | ESR-mediated signaling |
| *EBAG9* | 8 | 70 | 0.346 | 0.514 | 0.222 | ESR-mediated signaling |
| *AGO2* | 8 | 339 | 0.405 | 0.552 | 0.269 | ESR-mediated signaling |
| *POLR2K* | 8 | 21 | 0.686 | 0.634 | 0.672 | ESR-mediated signaling |
| *S1PR3* | 9 | 58 | 0.213 | 9.93×10^-2^ | 0.481 | ESR-mediated signaling |
| *GNG10* | 9 | 84 | 0.248 | 0.338 | 0.185 | ESR-mediated signaling |
| *KANK1* | 9 | 1336 | 0.250 | 0.508 | 0.125 | ESR-mediated signaling |
| *CDK9* | 9 | 31 | 0.256 | 0.420 | 0.156 | ESR-mediated signaling |
| *ZDHHC21* | 9 | 325 | 0.855 | 0.897 | 0.707 | ESR-mediated signaling |
| *GATA3* | 10 | 126 | 0.382 | 0.269 | 0.520 | ESR-mediated signaling |
| *CXCL12* | 10 | 105 | 0.790 | 0.732 | 0.756 | ESR-mediated signaling |
| *SMC3* | 10 | 122 | 0.864 | 0.863 | 0.705 | ESR-mediated signaling |
| *GPAM* | 10 | 126 | 0.986 | 0.981 | 0.951 | ESR-mediated signaling |
| *GNG3* | 11 | 13 | 0.205 | 0.330 | 0.133 | ESR-mediated signaling |
| *FDX1* | 11 | 131 | 0.232 | 0.290 | 0.192 | Pregnenolone biosynthesis |
| *H2AFX* | 11 | 17 | 0.280 | 0.368 | 0.210 | ESR-mediated signaling |
| *PGR* | 11 | 330 | 0.295 | 0.487 | 0.174 | ESR-mediated signaling |
| *CTSD* | 11 | 50 | 0.469 | 0.379 | 0.526 | ESR-mediated signaling |
| *POLR2L* | 11 | 48 | 0.560 | 0.422 | 0.650 | ESR-mediated signaling |
| *CCND1* | 11 | 16 | 0.648 | 0.547 | 0.704 | ESR-mediated signaling |
| *POLR2G* | 11 | 11 | 0.651 | 0.716 | 0.515 | ESR-mediated signaling |
| *MMP3* | 11 | 48 | 0.697 | 0.723 | 0.600 | ESR-mediated signaling |
| *KAT5* | 11 | 43 | 0.717 | 0.618 | 0.743 | ESR-mediated signaling |
| *HRAS* | 11 | 61 | 0.734 | 0.750 | 0.630 | ESR-mediated signaling |
| *MMP7* | 11 | 72 | 0.965 | 0.987 | 0.876 | ESR-mediated signaling |
| *PTGES3* | 12 | 66 | 2.35×10^-2^ | 1.50×10^-2^ | 0.101 | ESR-mediated signaling |
| *SP1* | 12 | 74 | 4.29×10^-2^ | 2.15×10^-2^ | 0.224 | ESR-mediated signaling |
| *CCNT1* | 12 | 79 | 9.60×10^-2^ | 4.82×10^-2^ | 0.268 | ESR-mediated signaling |
| *FKBP4* | 12 | 15 | 9.74×10^-2^ | 6.69×10^-2^ | 0.166 | ESR-mediated signaling |
| *H2AFJ* | 12 | 40 | 0.330 | 0.603 | 0.173 | ESR-mediated signaling |
| *KRAS* | 12 | 169 | 0.493 | 0.279 | 0.767 | ESR-mediated signaling |
| *GNB3* | 12 | 28 | 0.570 | 0.433 | 0.660 | ESR-mediated signaling |
| *CDKN1B* | 12 | 24 | 0.757 | 0.741 | 0.725 | ESR-mediated signaling |
| *GTF2F2* | 13 | 283 | 0.709 | 0.685 | 0.573 | ESR-mediated signaling |
| *FOS* | 14 | 40 | 0.124 | 0.193 | 9.34×10^-2^ | ESR-mediated signaling |
| *GTF2A1* | 14 | 97 | 0.166 | 0.353 | 8.77×10^-2^ | ESR-mediated signaling |
| *GNG2* | 14 | 411 | 0.446 | 0.420 | 0.410 | ESR-mediated signaling |
| *CALM1* | 14 | 46 | 0.496 | 0.407 | 0.565 | ESR-mediated signaling |
| *AKT1* | 14 | 44 | 0.503 | 0.370 | 0.619 |  |
| *FOXA1* | 14 | 38 | 0.532 | 0.458 | 0.531 | ESR-mediated signaling |
| *HSP90AA1* | 14 | 147 | 0.543 | 0.570 | 0.472 | ESR-mediated signaling |
| *YY1* | 14 | 99 | 0.609 | 0.755 | 0.434 | ESR-mediated signaling |
| *ESR2* | 14 | 260 | 0.849 | 0.734 | 0.887 | ESR-mediated signaling |
| *GNB5* | 15 | 215 | 0.296 | 0.136 | 0.627 | ESR-mediated signaling |
| *CYP19A1* | 15 | 343 | 0.328 | 0.466 | 0.219 | Oestrogen biosynthesis |
| *IGF1R* | 15 | 843 | 0.709 | 0.788 | 0.518 | ESR-mediated signaling |
| *TLE3* | 15 | 109 | 0.877 | 0.806 | 0.833 | ESR-mediated signaling |
| *CYP11A1* | 15 | 76 | 0.929 | 0.884 | 0.930 | Pregnenolone biosynthesis |
| *GTF2A2* | 15 | 78 | 0.944 | 0.936 | 0.886 | ESR-mediated signaling |
| *MMP2* | 16 | 116 | 5.36×10^-2^ | 4.48×10^-2^ | 0.101 | ESR-mediated signaling |
| *TNRC6A* | 16 | 189 | 0.110 | 0.117 | 0.142 | ESR-mediated signaling |
| *POLR2C* | 16 | 46 | 0.202 | 0.128 | 0.335 | ESR-mediated signaling |
| *AXIN1* | 16 | 259 | 0.230 | 0.315 | 0.174 | ESR-mediated signaling |
| *GNG13* | 16 | 35 | 0.253 | 0.212 | 0.302 | ESR-mediated signaling |
| *MAPK3* | 16 | 20 | 0.282 | 0.373 | 0.210 | ESR-mediated signaling |
| *CBFB* | 16 | 78 | 0.335 | 0.271 | 0.389 | ESR-mediated signaling |
| *ZDHHC7* | 16 | 219 | 0.463 | 0.415 | 0.484 | ESR-mediated signaling |
| *HSD17B2* | 16 | 116 | 0.722 | 0.610 | 0.703 | Oestrogen biosynthesis |
| *CREBBP* | 16 | 190 | 0.867 | 0.695 | 0.872 | ESR-mediated signaling |
| *MED1* | 17 | 90 | 3.14×10^-3^ | 2.62×10^-3^ | 3.45×10^-2^ | ESR-mediated signaling |
| *STARD3* | 17 | 89 | 3.78×10^-2^ | 6.91×10^-2^ | 3.82×10^-2^ | Pregnenolone biosynthesis |
| *POLR2A* | 17 | 155 | 4.63×10^-2^ | 0.200 | 2.14×10^-2^ | ESR-mediated signaling |
| *KPNA2* | 17 | 16 | 4.92×10^-2^ | 4.91×10-2 | 6.63×10^-2^ | ESR-mediated signaling |
| *FDXR* | 17 | 31 | 0.148 | 0.297 | 8.43×10^-2^ | Pregnenolone biosynthesis |
| *GNGT2* | 17 | 43 | 0.375 | 0.465 | 0.286 | ESR-mediated signaling |
| *SPHK1* | 17 | 60 | 0.567 | 0.619 | 0.469 | ESR-mediated signaling |
| *TSPOAP1* | 17 | 101 | 0.638 | 0.782 | 0.477 | Pregnenolone biosynthesis |
| *DDX5* | 17 | 35 | 0.656 | 0.659 | 0.577 | ESR-mediated signaling |
| *HSD17B1* | 17 | 24 | 0.943 | 0.946 | 0.871 | Oestrogen biosynthesis |
| *TNRC6C* | 17 | 181 | 0.958 | 0.971 | 0.894 | ESR-mediated signaling |
| *STARD6* | 18 | 99 | 0.102 | 0.125 | 0.112 | Pregnenolone biosynthesis |
| *BCL2* | 18 | 505 | 0.296 | 0.371 | 0.229 | ESR-mediated signaling |
| *POLR2I* | 19 | 23 | 0.145 | 0.259 | 8.88×10^-2^ | ESR-mediated signaling |
| *GNG8* | 19 | 24 | 0.147 | 0.100 | 0.232 | ESR-mediated signaling |
| *USF2* | 19 | 56 | 0.185 | 0.140 | 0.258 | ESR-mediated signaling |
| *PRMT1* | 19 | 20 | 0.224 | 0.214 | 0.239 | Oestrogen biosynthesis |
| *HSD17B14* | 19 | 51 | 0.256 | 0.233 | 0.283 | ESR-mediated signaling |
| *GNG7* | 19 | 543 | 0.258 | 0.200 | 0.339 | ESR-mediated signaling |
| *CARM1* | 19 | 73 | 0.332 | 0.266 | 0.392 | ESR-mediated signaling |
| *FDX2* | 19 | 46 | 0.479 | 0.594 | 0.343 | Pregnenolone biosynthesis |
| *PPP5C* | 19 | 125 | 0.571 | 0.404 | 0.705 | ESR-mediated signaling |
| *JUND* | 19 | 40 | 0.676 | 0.535 | 0.777 | ESR-mediated signaling |
| *FOSB* | 19 | 65 | 0.687 | 0.670 | 0.650 | ESR-mediated signaling |
| *KDM4B* | 19 | 554 | 0.692 | 0.984 | 0.437 | ESR-mediated signaling |
| *POLR2E* | 19 | 83 | 0.693 | 0.691 | 0.605 |  |
| *GTF2F1* | 19 | 79 | 0.727 | 0.580 | 0.790 | ESR-mediated signaling |
| *PIK3R2* | 19 | 54 | 0.729 | 0.882 | 0.511 | ESR-mediated signaling |
| *AKT2* | 19 | 113 | 0.872 | 0.940 | 0.726 | ESR-mediated signaling |
| *NCOA3* | 20 | 313 | 0.181 | 0.375 | 9.79×10^-2^ | ESR-mediated signaling |
| *SRC* | 20 | 131 | 0.287 | 0.431 | 0.188 | ESR-mediated signaling |
| *ZNF217* | 20 | 59 | 0.508 | 0.374 | 0.601 | ESR-mediated signaling |
| *MMP9* | 20 | 52 | 0.802 | 0.776 | 0.734 | ESR-mediated signaling |
| *RUNX1* | 21 | 574 | 9.01×10^-3^ | 2.40×10^-2^ | 2.14×10^-2^ | ESR-mediated signaling |
| *H2BFS* | 21 | 22 | 4.84×10^-2^ | 0.152 | 2.40×10^-2^ | ESR-mediated signaling |
| *TFF3* | 21 | 34 | 7.39×10^-2^ | 0.348 | 2.48×10^-2^ | ESR-mediated signaling |
| *NRIP1* | 21 | 193 | 0.546 | 0.443 | 0.608 | ESR-mediated signaling |
| *TFF1* | 21 | 78 | 0.573 | 0.349 | 0.785 | ESR-mediated signaling |
| *EP300* | 22 | 129 | 1.70×10^-2^ | 7.29×10^-3^ | 0.145 | ESR-mediated signaling |
| *MAPK1* | 22 | 245 | 9.35×10^-2^ | 9.19×10^-2^ | 0.144 | ESR-mediated signaling |
| *POLR2F* | 22 | 29 | 0.112 | 0.116 | 0.142 | ESR-mediated signaling |
| *TSPO* | 22 | 66 | 0.363 | 0.560 | 0.224 | Pregnenolone biosynthesis |
| *TNRC6B* | 22 | 406 | 0.454 | 0.594 | 0.310 | ESR-mediated signaling |

The three models used are: 1) SNP-wise Mean, 2) SNP-wise Top 1 and 3) Multi model. The two SNP-wise models examine the individual SNPs present in the gene and subsequently combine the resulting P-values of the SNPs into a gene test statistic, while the multi model runs the basic models (SNP-wise) and combines the resulting P-values into an aggregated P-value for the gene.

**Supplementary table S4.** Results of gene set analysis performed with MAGMA software.

| Pathway | N° of genes | Beta | Standard Error | P-value |
| --- | --- | --- | --- | --- |
| Pregnenolone biosynthesis | 12 | 0.198 | 0.288 | 0.246 |
| Oestrogen biosynthesis | 6 | -0.017 | 0.403 | 0.517 |
| ESR-mediated signaling | 190 | -0.152 | 0.237 | 0.739 |
